# Different Kinetics of Serum ADAMTS13, GDF-15, and Neutrophil Gelatinase-Associated Lipocalin in the Early Phase of Aneurysmal Subarachnoid Hemorrhage

**DOI:** 10.3390/ijms241311005

**Published:** 2023-07-02

**Authors:** Peter Csecsei, Csaba Olah, Reka Varnai, Diana Simon, Szabina Erdo-Bonyar, Timea Berki, Mate Czabajszki, Laszlo Zavori, Attila Schwarcz, Tihamer Molnar

**Affiliations:** 1 Department of Neurosurgery, Medical School, University of Pecs, 7624 Pecs, Hungary; csecsei.peter@pte.hu (P.C.); schwarcz.attila@pte.hu (A.S.); 2 Neurosurgical Unit, B.A.Z. County Hospital, 3526 Miskolc, Hungary; olahcs@gmail.com (C.O.); czamate@gmail.com (M.C.); 3 Department of Primary Health Care, Medical School, University of Pecs, 7624 Pecs, Hungary; 4 Department of Immunology and Biotechnology, Medical School, University of Pecs, 7624 Pecs, Hungary; simon.diana@pte.hu (D.S.); erdo-bonyar.szabina@pte.hu (S.E.-B.); berki.timea@pte.hu (T.B.); 5 Emergency Department, Saudi German Hospital, Dubai 391093, United Arab Emirates; zavori.laszlo@gmail.com; 6 Department of Anaesthesiology and Intensive Care, Medical School, University of Pecs, 7624 Pecs, Hungary; tihamermolnar@yahoo.com

**Keywords:** subarachnoid hemorrhage, GDF-15, neutrophil gelatinase-associated lipocalin, ADAMTS13, delayed cerebral ischemia, macrovascular vasospasm

## Abstract

Growth differentiation factor 15 (GDF-15), neutrophil gelatinase-associated lipocalin (NGAL), and ADAMTS13 have previously been implicated in the pathophysiological processes of SAH. In the present study, we aim to examine their role in the early period of SAH and their relationship to primary and secondary outcomes. Serum samples were collected at five time periods after SAH (at 24 h (D1), at 72 h (D3), at 120 h (D5), at 168 h (D7) and at 216 h (D9), post-admission) and) were measured by using MILLIPLEX Map Human Cardiovascular Disease (CVD) Magnetic Bead Panel 2. We included 150 patients with SAH and 30 healthy controls. GDF-15 levels at D1 to D9 were significantly associated with a 3-month unfavorable outcome. Based on the ROC analysis, in patients with a good clinical grade at admission (WFNS I-III), the GDF-15 value measured at time point D3 predicted a 3-month unfavorable outcome (cut-off value: 3.97 ng/mL, AUC:0.833, 95%CI: 0.728–0.938, sensitivity:73.7%, specificity:82.6%, *p* < 0.001). Univariate binary logistic regression analysis showed that serum NGAL levels at D1-D5 and ADAMTS13 levels at D7-D9 were associated with MVS following SAH. GDF-15 is an early indicator of a poor 3-month functional outcome even in patients with mild clinical conditions at admission.

## 1. Introduction

Subarachnoid hemorrhage (SAH) is caused by cerebral aneurysm rupture in approx. 5% of all strokes but is associated with exceptionally high mortality and severe residual symptoms [1]. Early brain injury (EBI) results from pathological mechanisms triggered by SAH including microthrombosis and reactive inflammation, which collectively contribute to neuronal damage subsequent to SAH. Complications, such as the occurrence of delayed cerebral ischemia (DCI) or cerebral vasospasm greatly influence the outcome [2]. Cerebral vasospasm occurs in approximately 70% of cases, while delayed cerebral ischemia develops in about 20–30% of all cases. The diagnosis is based on the deterioration of neurological status and the detection of vasospasm through transcranial ultrasound examination or imaging modalities such as CT or MR angiography, and DSA [2]. Their pathophysiology is multifaceted [3]. Strategies directed toward minimizing early brain injury hold the best promise for further reducing mortality after SAH [3]. The key components involved in the thromboinflammatory cascade present potential targets for therapeutic intervention, thereby offering a novel approach to ameliorate the extent of neuronal injury following SAH. The following thromboinflammatory molecules were examined in this study: (i) growth differentiation factor 15 (GDF-15), a stress-responsive member of the transforming growth factor cytokine superfamily, a marker of cardiovascular events [4], that shows associations with inflammation [5] and cerebral injury caused by ischemic stroke [6,7,8]. Furthermore, GDF-15 was proved to be a predictor of neurological improvement and long-term outcomes after thrombectomy [9]. GDF-15 was also found to be correlated with vascular brain injury and increased risk of incident dementia [10]. Recently, high circulating GDF-15 levels were associated with incident ICH and SAH [11]. Moreover, it may mediate iron toxicity in hemorrhagic stroke models [12] (ii) Lipocalin-2, also known as neutrophil gelatinase-associated lipocalin (NGAL), is an important mediator of neuroinflammation in vascular brain injury [13]. In addition, a recent study with a modest sample size examined NGAL on both sides of the blood–brain barrier in SAH and found an association between NGAL and SAH outcome [14]. (iii) ADAMTS13 is a von Willebrand factor-cleaving protease that inhibits thrombus formation resulting in reduced neuronal injury after SAH [15,16]. Increasing evidence supports the involvement of platelet aggregation in EBI and the development of DCI [17]. Furthermore, a recent study found lower levels of ADAMTS13 in patients with SAH complicated with DCI [18]. We hypothesized that persistent systemic inflammation and specific mechanisms impacting thrombus formation play significant roles in the development of EBI. Therefore, the aim of our study was to explore the temporal profile of serum levels of ADAMTS13, GDF-15, and NGAL and their relations to primary (3-month clinical outcome) and secondary (macrovascular vasospasm and delayed cerebral ischemia) outcomes. We also wanted to determine the relationship among the three markers in the early phase of SAH.

## 2. Results

### 2.1. Patients’ Characteristics

Table 1 summarizes the baseline characteristics of the SAH (*n* = 150) cohort. Initially, 211 subjects were screened. Drop out: perimesencephalic hemorrhage (*n* = 8), hemorrhage due to arteriovenous malformation (*n* = 3), SARS-CoV-2 positivity on admission (*n* = 3), hospital admission later than 24 h after ictus (*n* = 9), systemic diseases (n = 11), lost to follow-up (*n* = 12), unavailable biomarker measurements (*n* = 8), death before D9 (*n* = 7). The mean age of the population was 55 ± 12 years and almost half of the patients had hypertension (47%). Aneurysms were treated with endovascular coiling only. Among the patients admitted with more serious bleeding (mFisher score III-IV, *n* = 99), there were more who had macrovascular vasospasm (34% vs. 12%, *p* = 0.019), DCI (23% vs. 0%, *p* = 0.005) and required external ventricular drainage (58% vs. 18%, *p* < 0.001) than among those admitted with minor bleeding (mFisher score I-II, *n* = 51). Patients with the unfavorable 3-month outcome (*n* = 80, mRankin score = 3–6) were older (58 ± 12 vs. 52 ± 12, *p* < 0.001) and had a higher admission serum CRP (27 mg/dL, (IQR 9–73) vs. 8 (3–17), *p* = 0.002) and NLR value (6 (4–11) vs. 4 (3–7), *p* = 0.002) and more patients were in serious condition (WFNS IV-V) on admission than those with the favorable 3-month outcome (unfavorable: 45 vs. favorable: 2). Those with multiple aneurysms (*n* = 43) had macrovascular vasospasm more often (49% vs. 20%, *p* = 0.001) than those with solitary aneurysms. The median serum level of ADAMTS13, GDF-15, and NGAL at D1 was as follows: 2516 (1826–2981) ng/mL, 3.5 (2–5) ng/mL, and 490 (281–720), respectively. The characteristics of the SAH cohort (age, gender distribution, and severity) are similar to those described in previous large SAH studies [19,20]. The median age of the control cases was 58 years (interquartile range [IQR] 49–66), and 58.4% were women.

### 2.2. Levels of ADAMTS13, GDF-15, and NGAL in Different SAH Severity Groups

The median serum (ng/mL) values (IQR) of markers in patients and controls were as follows: ADAMTS13: 2520 (1911–3051) vs. 2990 (2462–3878), *p* = 0.014, GDF-15: 3.4 (2–5) vs. 1.8 (1–2.6), *p* < 0.001 and NGAL: 411 (266–677) vs. 517 (379–615), *p* = 0.255. Table 2 presents the serial changes in the levels of each biomarker in the patients with SAH according to different clinical and radiological severity. The serum GDF-15 level was significantly higher in the high-grade (WFNS IV-V) group compared to the low-grade (WFNS I-III) patients at all time points. The levels of all three biomarkers did not differ significantly with time (P_trend_ > 0.05), Table 2. When stratified according to modified Fisher grade, patients with high-grade bleeding (mFisher score = III-IV) had a significantly higher level of ADAMTS13 at D1 and D3 compared with that of patients with low-grade hemorrhage (mFisher score = I-II). The serum level of NGAL at D1 and D3 was significantly higher in the group with high-grade bleeding than in those with low-grade hemorrhage.

### 2.3. Association of ADAMTS13, NGAL, and GDF-15 with 3-Month Outcome

**Figure 1:**
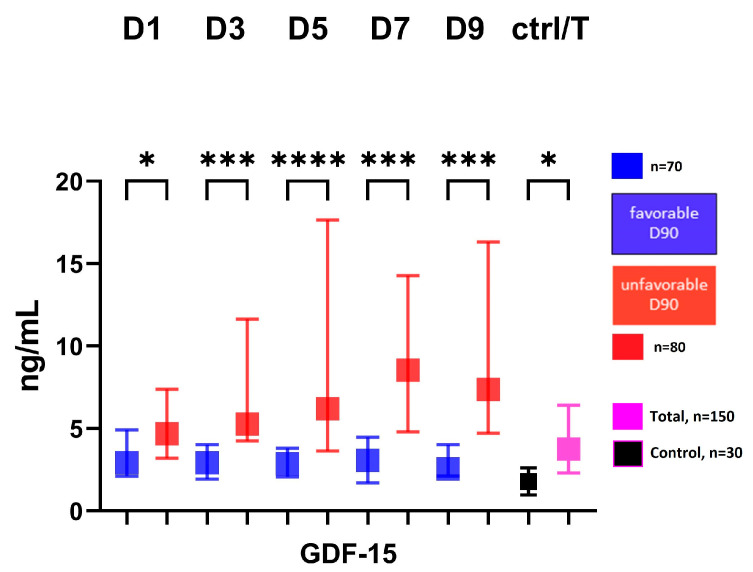
Serum level of GDF-15 in patients according to 3-month outcome. Favorable group defined as modified Rankin score: 0–2 at 3-month follow-up; unfavorable group defined as: modified Rankin score 3–6 at 3-month follow-up; GDF-15, growth differentiation factor-15; D, day; ctrl, control; T, total number of subjects; * *p* < 0.05, *** *p* < 0.001, **** *p* < 0.0001.

The serum GDF-15 level showed a significantly higher level at all time points in the group with an unfavorable 3-month outcome (modified Rankin score: 3–6) compared to the group with a favorable 3-month outcome (modified Rankin score: 1–2), Figure 1.

The full set of all three biomarker measurements and clinical variables dichotomized according to the 3-month outcome, are shown in Table S1. Serum levels of NGAL (ng/mL) measured on D5 and D7 are slightly higher in the unfavorable 3-month outcome group, but the significance is weak (D5; favorable: 304 IQR [223–538] vs. unfavorable: 380 [316–705], *p* = 0.049; D7; favorable: 382 [253–539] vs. unfavorable: 513 [313–698], *p* = 0.03). Serum ADAMTS13 level showed no correlation with the 3-month outcome at any time point. The levels of all three biomarkers did not differ significantly with time (*p* trend > 0.05), Table S1. In univariable logistic regression, GDF-15 levels at D1 to D9 were significantly associated with a 3-month unfavorable outcome. These associations remained significant after adjustment for well-established predictors of SAH-related unfavorable outcomes (age, gender, WFNS, DCI during hospitalization) in a binary logistic regression model, Table 3.

Based on the ROC analysis, the GDF-15 value measured at time points D3-D5-D7-D9 indicates an unfavorable 3-month outcome with excellent sensitivity and specificity, but if we take patients with a good clinical grade at admission (WFNS I-III), only the GDF-15 value measured at time point D3 can make a similar prediction (cut-off value: 3.97 ng/mL, AUC:0.833, 95%CI: 0.728–0.938, sensitivity:73.7%, specificity:82.6%, *p* < 0.001), Table 4.

### 2.4. NGAL and ADAMTS13 in Patients with and without Macrovascular Vasospasm

**Figure 2:**
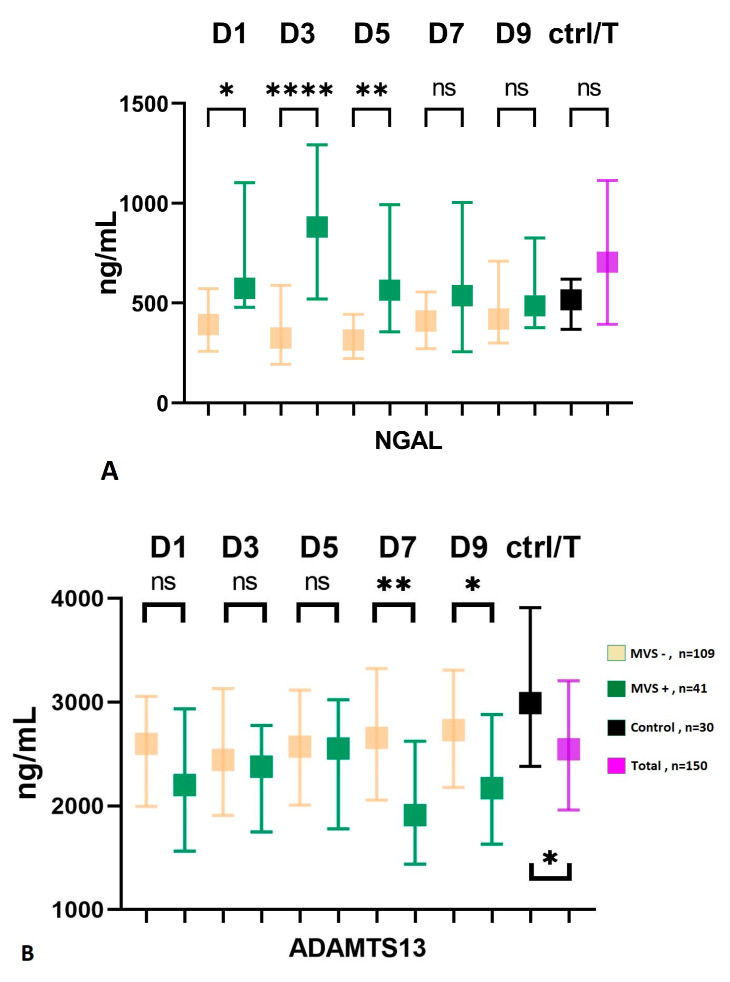
Serum level of NGAL (**A**) and ADAMTS13 (**B**) in patients with macrovascular vasospasm. MVS, macrovascular vasospasm; D, day; ADAMTS13, a disintegrin and metalloproteinase with a thrombospondin type 1 motif; member, 13; NGAL, neutrophil gelatinase-associated lipocalin; ctrl, control; T, total number of subjects; ns = non-significant; * *p* < 0.05, ** *p* < 0.01, **** *p* < 0.0001.

Although not statistically significant, subjects with severe conditions at admission (WFNS IV-V) exhibited a higher incidence of macrovascular vasospasm compared to patients with low-grade SAH (WFNS I-III), with frequencies of 25% and 31%, respectively (*p* = 0.425). Similarly, the group with high-grade bleeding at admission had a higher frequency of MVS than the low-grade group, and this association was statistically significant (12% vs. 34%, *p* = 0.015). The group of patients with macrovascular vasospasm demonstrated a significantly poorer 3-month outcome compared to the MVS-negative group (mRS score, 4 (IQR: 2–5) vs. 2 (1–5), *p* = 0.002). Additionally, the presence of smokers was more prevalent in the MVS-positive group (14% vs. 31%, *p* = 0.021), and neutrophil counts (G/L) upon admission were also higher in this group with a median of 11 (IQR: 8–14) compared to 9 (IQR: 7–12) in the MVS-negative group (*p* = 0.007). Serum levels of GDF-15 did not show a correlation with macrovascular vasospasm in the present cohort. In the early phase (D1-D3-D5), the serum level of NGAL was significantly higher in the group affected by macrovascular vasospasm (Figure 2A), while in the late phase (D7-D9), the serum level of ADAMTS13 showed significantly lower levels in patients with MVS, Figure 2B.

ROC curve analyses were performed and demonstrated that NGAL at D3 was able to distinguish patients with MVS from patients without MVS with acceptable power (cut-off value: 518 ng/mL, AUC:0.776, 95%CI: 0.665–0.887, sensitivity: 78.3%, specificity: 73.1%, *p* < 0.001), Table 5.

Univariate binary logistic regression analysis showed that serum NGAL level at D1-D5 and ADAMTS13 level at D7-D9 were associated with macrovascular vasospasm following SAH, Table 6. Binary logistic regression analysis of patients with SAH, including all significant variables (age, gender, mFisher score at admission CT, systemic infection during hospitalization), only identified ADAMTS13 at D7 as independent predictors of macrovascular vasospasm (OR 0.999, 95%-CI: 0.997–1.000, *p* = 0.036).

### 2.5. Association of Serum Levels of NGAL and GDF-15 in Patients with and without DCI

**Figure 3:**
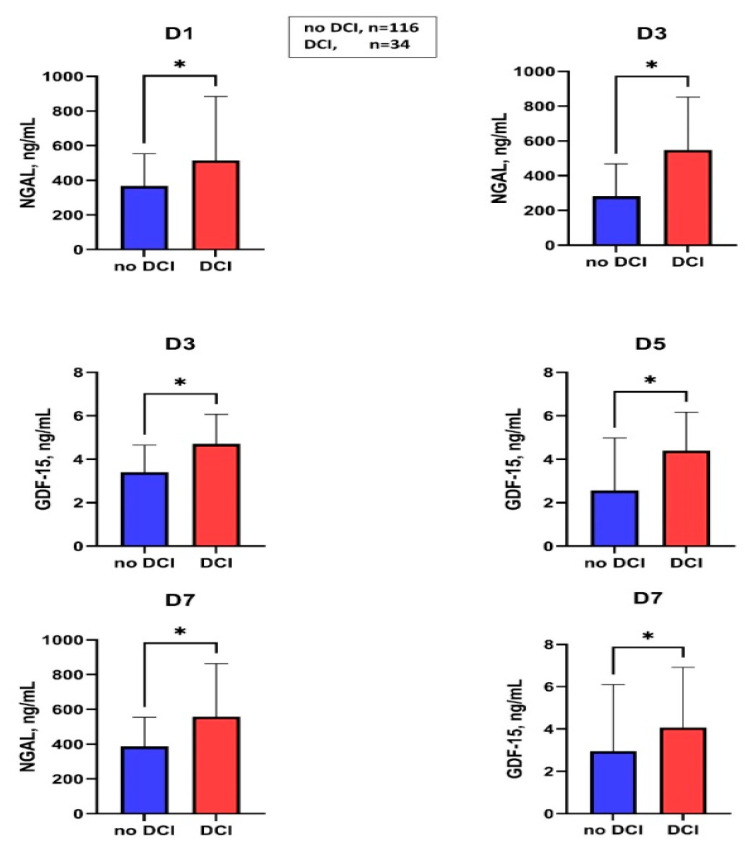
Serum levels of NGAL and GDF-15 in patients with and without DCI at different sampling times. DCI, delayed cerebral ischemia; D, day; NGAL, neutrophil gelatinase-associated lipocalin; GDF-15, growth differentiation factor-15; * *p* < 0.05; the values of NGAL on days D5 and D9 and the values of GDF-15 on days D1 and D9 were not shown, as they did not show significant differences between the DCI positive and DCI negative groups.

Figure 3 summarizes the markers associated with DCI. In the case of DCI in the group without macrovascular vasospasm, a significantly higher GDF-15 level was observed at D3 compared to DCI-negative subjects (no-DCI: 3.4 (1.8–4.6) vs. DCI: 5.5 (4.7–14.4), *p* = 0.02), Table S2, however in binary logistic regression analysis (included all confounding factors, such as modified Fisher score, age, the occurrence of systemic infection during hospitalization) it was not proved to be an independent predictor of DCI in patients with macrovascular vasospasm during hospitalization after aneurysmal SAH.

### 2.6. Correlation of Biomarkers with Each Other and Admission Inflammatory Parameters

**Figure 4:**
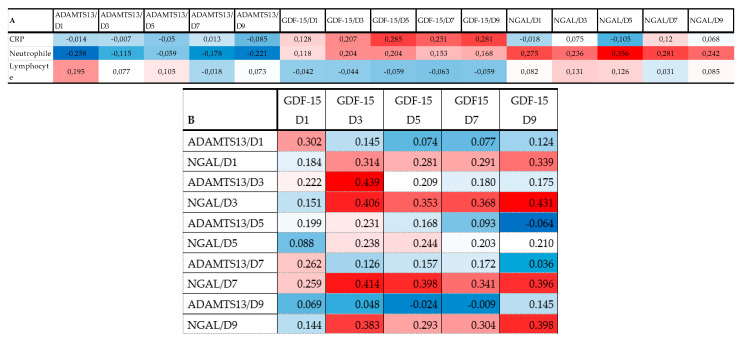
Heat map of correlation analysis among admission inflammatory variables and thromboinflammatory molecules ADAMTS13, GDF-15, and NGAL (**A**) and thromboinflammatory molecules with each other (**B**). (note: red indicates that the two parameters were positively correlated, and blue indicates that the two parameters were negatively correlated. The darker the color, the stronger the correlation).

Figure 4A represents a heatmap with Spearman correlation coefficients calculated for admission inflammatory markers plus ADAMTS13, GDF-15, and NGAL (all D1-D9). The serum level of GDF-15 showed a positive correlation at D5-D7-D9 time points with CRP. The heatmap also confirmed a positive correlation between neutrophile count at admission and the level of NGAL at all time points (D1-D9). For more correlations see Figure 4A.

Heatmaps of Spearman correlation analysis. The correlation heatmap is used to represent significant statistical correlation values (r) between ADAMTS13, GDF-15, and NGAL measured at different time points (D1: day 1; D3 = day 3; D5 = day 5; D7 = day 7; D9 = day 9) and inflammatory markers at admission (A). Thromboinflammatory molecules were also correlated with each other (B). In the heatmap, red indicates a positive, while blue indicates a negative correlation. The darker the color, the stronger the correlation. The Spearman rank correlation test was used. A *p*-value < 0.05 was accepted as significant. *p* > 0.10 are presented in white squares.

## 3. Discussion

### 3.1. GDF-15 and Outcome

In this study, the following main findings were observed during the analysis of the GDF-15 serum levels and outcome endpoints: (i) The serum level of GDF-15 was significantly higher in the patient group compared to the control group, (ii) a significantly higher level was observed in patients displaying a high-grade clinical grade compared to those with a low-grade condition on admission, (iii) the GDF-15 serum level at each sampling time (D1-D9) was an independent predictor of the unfavorable outcome at 3 months, (iv) among patients in a low-grade state on admission, elevated serum levels of GDF-15 in the early stage (D3) indicated a later unfavorable 3-month outcome, and finally (v) the level of GDF-15 showed a positive correlation with admission C-reactive protein levels during all time points except D1. The level of GDF-15 on D1 showed a lower correlation with the outcome than the values measured on D3 and D5, which suggests that the processes influencing its level became more pronounced after the first day. In a previous study, the GDF-15 level was significantly elevated in patients after ischemic stroke with poor outcomes [7], however, the degree of brain damage reflected by the NIHSS score on admission was not severe. Moreover, the level of GDF-15 decreased after 24 h compared to admission levels. The same decrease was found in another cohort of ischemic stroke patients between admission and day 7 [13]. These findings suggest that in the initial days following SAH, there is a continuous occurrence of processes that lead to an elevation in levels of GDF-15. This stands in contrast to ischemic stroke, where the peak of brain tissue damage resulting from vessel occlusion is typically observed within the first 24 h.

Our results further support this observation by demonstrating that elevated levels of GDF-15 measured in patients with a low-grade clinical state upon admission and during the later phase of the disease signify the presence of ongoing processes that have detrimental effects on the subsequent outcome. Our results emphasize the importance of early aggressive treatment even in patients in good condition, so that the effect of EBI can be minimized. GDF-15 can act on neighboring cells regulating inflammatory interactions, in autocrine, paracrine, and endocrine manners [21] and triggers an adaptive response of tissular protection, with emphatic antiproliferative, anti-inflammatory, and anti-apoptotic actions [22], yet it is unclear whether it leads to the further progression of the disease or provides protection against the disease [23]. This phenomenon can be partially explained by the regulatory role of GDF-15 in modulating the immune response of the host to various infectious and non-infectious diseases. GDF-15 has the capability to inhibit the type 1 inflammatory response while concurrently promoting the type 2 inflammatory response, as evidenced by previous studies [24]. Worthmann et al. suggested that the circulating levels of GDF-15 integrate information from different disease pathways that are relevant for stroke outcomes, such as cardiovascular disease burden, brain damage, and inflammation [6]. Our results highlight the close relationship of GDF-15 with inflammation, as its levels (D3-D9) showed a positive correlation with admission CRP and with the level of NGAL at D3 to D9, however, we cannot clearly identify the source of GDF-15. Previous research has demonstrated that C-reactive protein (CRP) can induce the expression of GDF-15 mRNA and protein levels in human aortic cell cultures, and this effect is mediated by the presence of p53 [25]. Another study has also suggested a potential association between GDF-15 and CRP [26]. However, it is important to note that although the levels of these two biomarkers may fluctuate together, it does not necessarily imply a shared role in the underlying pathomechanisms. Higher circulating concentrations of GDF-15 were associated with both incident ICH and incident SAH in a nested case–control study, but samples were not collected after hemorrhagic stroke [5]. The mentioned study had a limited scope as it solely encompassed a single sampling time prior to the occurrence of stroke, without investigating the association between GDF-15 levels and the subsequent outcome. Outside the reproductive organs, GDF-15 shows low or absent constitutive expression; however, it can be induced in many cell types under stress conditions, making the source of the high serum level difficult to determine [27]. In summary, GDF-15 is a sensitive and specific marker of unfavorable outcomes, especially in the early stages of SAH, even among patients with mild symptoms. Despite the detected increase in GDF-15 levels in our study, the specific source of this elevation remains unclear. Further comprehensive investigations are warranted to explore the underlying mechanisms and determine the precise origins of GDF-15 in the context of our findings.

### 3.2. NGAL and ADAMTS13 in Macrovascular Vasospasm

Based on the additional results of our study, NGAL in the early phase particularly on the 3rd post-SAH day showed a correlation with the later appearance (predominantly on the 8th day) of macrovascular vasospasm, but it did not prove to be an independent predictor. In addition, it showed a positive correlation with the admission neutrophile count and with the level of NGAL at the sampling times D3-D9. NGAL stimulates neutrophil migration and neutrophilic cytokines production including IL-6, TNF-a, and IL-1b, and elevated blood NGAL is associated with systemic inflammation [28]; furthermore, NGAL binds and stabilizes human matrix metallopeptidase-9 (MMP-9) which regulates neutrophils in inflammatory processes [29]. In a small SAH cohort by Yu et al. found that CSF NGAL levels correlated with the outcome with low significance, whereas plasma NGAL levels did not and plasma NGAL levels did not differ from controls [14]. This corresponds to the results observed in our study. Another similarity is that the NGAL level measured in the plasma correlated with the admission mFisher score, which we also observed in the first three days after the SAH. An interesting observation in this study was the close correlation observed between NGAL levels measured on days 3 and 5 with plasma IL-6/MMP-9 and TNFalpha levels. In our study, we similarly found a positive correlation between NGAL and macrovascular vasospasm (MVS) on these particular days. However, Yu et al. could not demonstrate a correlation between CSF or plasma NGAL levels and angiographic vasospasm. In the rat ICH model [30], the level of NGAL begins to rise early after the stroke and continues to increase until the 7th day, which was particularly pronounced in the perihematomal area. This and another study [31] also indicate a role of iron in regulating NGAL expression following ICH. In our study, its close correlation with the Fisher score, i.e., with the size of the bleeding, on days 1, 3, and 5 may also show its close correlation with the metabolism of the iron released during SAH, and this correlation weakens as the amount of bleeding decreases. However, NGAL may have multiple cerebral and peripheral sources. Astrocytes [30], infiltrating [32] and activated neutrophils [33], white matter injury [34] as well as other inflammatory conditions [35] may also be responsible for its upregulation. Its close association with bleeding is also confirmed by the study of Ni et al., where they found that both ICH and intracerebral iron injection caused upregulation of NGAL in mouse brain and ICH/iron-induced brain injury was less in NGAL knockout mice [36]. In our study, NGAL showed a close positive correlation with admission neutrophil count during the entire study. MMP-9 is increasingly expressed in a parallel time course to the development of cerebral vasospasm in this rat experimental model of SAH [37] and human studies also confirmed its potential role as a biomarker for cerebral vasospasm [38,39]. Il-6 [40] as well as TNFα [41] are also involved in the development of cerebral vasospasm. We found that the median (IQR) time to onset of macrovascular vasospasm was 8 (6–9) days after SAH so higher NGAL levels are an early indicator of subsequent vasospasm. However, it is not clear whether the elevated NGAL level itself participates in the development of vasospasm or only induces other processes leading to vasospasm.

In our study, the lower ADAMTS13 level detected on days 7 and 9 was associated with more frequent MVS. ADAMTS13 is a key protein in linking thrombosis with inflammation [11] and administration of ADAMTS13 after SAH produced significant amelioration of microthrombosis [7]. Deficiency of ADAMTS13 is associated with the occurrence of microthrombosis followed by ischemic complications, such as cerebral infarction [42]. ADAMTS13 shows a close correlation with DCI, as well as with micothromboses [43] involved in its pathogenesis, but no such correlation was found in our study. Considering that DCI is a multifactorial phenomenon [43] and microthrombosis is only one part of it, it can be assumed that the reduced ADAMTS13 activity in our patients with vasospasm is not related to DCI. As previously mentioned, NGAL stimulates neutrophil migration and the production of neutrophil cytokines, including IL-6, which is known to significantly reduce ADAMTS13 levels. Thus, the increase in NGAL levels detected in the early phase in our patients with vasospasm is accompanied by a consequent, late ADAMTS13 level decrease which may be caused by high NGAL expression. Nevertheless, in our study, we only found a correlation between ADAMTS13 and vasospasm, not between ADAMTS13 and DCI. In our study, we did not detect a correlation with the ADAMTS13 level in the early phase of SAH. In a recent study, it was found that ADAMTS13 activity decreases in the early phase of trauma [44]. Systemic inflammation may impact the activity and regulation of ADAMTS13; additionally, platelet activation can result in the release of von Willebrand factor and decreased levels of ADAMTS13 [44]. Both mechanisms play a significant role in the occurrence of early brain injury following aneurysmal subarachnoid hemorrhage (aSAH) [45]. However, our study did not establish a clear correlation between ADAMTS13 and early stage pathophysiological processes in aSAH and traumatic brain injury (TBI), despite their similarities. Several potential factors may account for this disparity. Firstly, our study focused on analyzing the serum level of ADAMTS13, whereas other studies [44] have examined ADAMTS13 activity. Additionally, we did not assess the level of von Willebrand Factor, which plays a crucial role in coagulation processes. Furthermore, the onset and duration of cerebral vasospasm differ between TBI and aSAH, indicating the involvement of alternative pathophysiological mechanisms [46].

In conclusion, in our study, we found that GDF-15 predicts subsequent unfavorable 3-month outcomes after SAH early with high sensitivity and specificity. This correlation also exists in patients with low-grade status on admission. NGAL exhibits a significant association with macrovascular vasospasm during the early phase of subarachnoid hemorrhage (SAH), whereas ADAMTS13 appears to be more closely related to this condition during the late phase of SAH. The correlation of the examined markers with DCI is lower than expected, especially in the case of ADAMTS13. GDF-15 shows a close positive correlation with NGAL and admission CRP levels, while NGAl shows a similar correlation with admission neutrophil count.

We propose that GDF-15 should undergo further investigations as a potential marker for risk stratification in patients with SAH.

### 3.3. Limitations

Our study has several important limitations. In our study, the levels of cardiac biomarkers, specifically troponin T, were not examined. However, it is important to acknowledge that all three investigated biomarkers can potentially be influenced by cardiac events. In the case of GDF-15, the observed correlations were strong, and there were no major cardiac events in the patient group, so we consider the absence of cardiac markers acceptable for the interpretation of the results. We only recorded admission inflammatory parameters, serial measurements were not performed, so we could not correlate our results with the inflammatory parameters at the individual measurement times. Only serum samples were collected, no CSF sample was taken, so we could not compare the expression of markers in serum and CSF. Finally, relatively few healthy controls were included in the study, and the follow-up period for patients was limited to 3 months.

## 4. Materials and Methods

### 4.1. Subjects and Study Design

This is a multi-center, ongoing prospective cohort of consecutive SAH patients admitted to the Department of Neurosurgery of the University of Pecs, Pecs, Hungary, and to the Neurosurgical Unit of B.A.Z. County Hospital, Miskolc, Hungary between December 2020 and December 2022. Institutional review board approval was obtained previously (IV/8468-1/2021/EKU), and written informed consent was obtained from each patient or their legal representative. Inclusion criteria were: age >18 years, spontaneous aneurysmal SAH diagnosed by computed tomography (CT) within 24 h of ictus, and aneurysm detected on digital subtraction angiography (DSA). Exclusion criteria were: traumatic SAH, pregnancy, hospital admission later than 24 h after ictus, no aneurysm treatment, bleeding from arteriovenous malformation, absence of a signed consent form, underlying SARS-CoV-2 infection, and systemic diseases (chronic neurological disease, tumors, liver and/or renal insufficiency, and chronic lung disease). Following the diagnosis of SAH, according to our hospital standards, the aneurysm was treated endovascularly within 24 h. In all instances, the patients were admitted to the neurointensive care unit for a minimum duration of 12–14 days, allowing for timely detection of anticipated complications, such as DCI. All patients received nimodipine 6 × 60 mg per os from the first day for vasospasm prevention. Routine TCD was performed on the patients daily. If macrovascular vasospasm and/or DCI occurred based on the TCD measurement or the deterioration of the clinical status (the occurrence of focal neurological impairment, or a decrease of at least 2 points on the Glasgow Coma Scale) [47], MR and MR angiography were performed to confirm macrovascular vasospasm on large vessels (ICA, MCA M1-M2, ACA, VA, BA) or DWI lesions. If the MR was not informative or was not clear, we performed a DSA and, if necessary, administered intra-arterial nimodipine. The diagnosis of delayed cerebral ischemia was confirmed if recent ischemia could be confirmed on the CT/MR before discharge compared to the diagnostic imaging. At the same time, 30 age- and gender-matched healthy volunteers were assigned to the healthy control group.

### 4.2. Clinical Definitions Data Collections and Outcome Measures

Clinical data collected included age, sex, medical and social history (hypertension, diabetes, smoking), and neurological status at admission based on the World Federation of Neurosurgical Societies scale (WFNS grade) from each patient. Each CT scan was assessed independently by a neurologist involved in the study, who evaluated the extent and distribution of blood based on the modified Fisher score (mFS) categorization. The low-grade group was defined as follows: WFNS I-III on admission clinical examination or mFisher score I-II at first CT scan. The high-grade group was defined as WFNS IV-V or mFisher score III-IV on admission evaluation. Functional outcome was assessed using the 0–6 modified Rankin scale (mRS). The 3-month mRS was obtained via a follow-up phone call by a trained research fellow using a standardized questionnaire or through a personal interview during the control DSA examination. Unfavorable outcome at 3 months after discharge was defined as a mRS of 3–6. Subjects were treated according to standard guidelines. Sonologic cerebral vasospasm was confirmed by TCD according to two criteria: mean velocities in the middle cerebral arteries >120 cm/s and Lindegaard ratios >3. Macrovascular vasospasm (MVS) was defined as narrowing of the arterial vessel lumen at MR or cerebral angiography. Presence of MVS was assessed by visual inspection of cerebral angiography or source images of cerebral angiography. The diagnosis had to be confirmed by an independent observer (neuroradiologist). Delayed cerebral ischemia (DCI) was diagnosed according to the previously proposed criteria [47]. DCI was diagnosed only after rigorous exclusion of other possible causes, and it was judged by the consensus of at least two neurointensivists.

The definition criteria of systemic infection were as follows: symptoms of infection with fever, elevated C-reactive protein and/or procalcitonin, and a positive diagnostic test such as chest X-ray or urine test. The primary outcome of this study was the occurrence of an unfavorable outcome at the 3-month mark, while secondary outcomes involved assessing the occurrence of DCI and the development of MVS.

### 4.3. Sample Collection and Processing Protocol

Serum samples were collected at four predetermined time points after ictus: 24 h (D1), 72 h (D3), 120 h (D5), 168 h (D7), and 216 h (D9). The samples were stored at −80 °C until measurements. The serum concentration of ADAMTS13, GDF-15, and Lipocalin-2/NGAL was determined using MILLIPLEX Map Human Cardiovascular Disease (CVD) Magnetic Bead Panel 2 (HCVD2MAG-67K, Merck KGaA, Darmstadt, Germany) according to the manufacturer’s recommendation. Briefly, 100-fold diluted serum samples, standards, and controls with equal volumes of assay buffer and fluorescent-coded magnetic bead mixture coated with capture antibodies specific for ADAMTS13, GDF-15, and Lipocalin-2/NGAL were placed in the corresponding wells of the 96-well plate. After overnight incubation, detection of bound analytes was performed with biotinylated detection antibody and streptavidin–phycoerythrin conjugate. The assay was run with Luminex MAGPIX instrument (Luminex Corporation, Austin, TX, USA). Data were analyzed with Belysa Immunoassay Curve Fitting Software, Version 1.2 (Merck KGaA, Darmstadt, Germany). Serum levels of lymphocyte, neutrophile, and C-reactive protein (CRP) were measured by standard detection methods from admission samples.

### 4.4. Statistical Analysis

Categorical variables are presented as numbers and percentages, while continuous variables are presented as medians and IQRs. Two-group comparisons were performed using the Mann–Whitney U test (continuous variables) or the χ^2^ test (categorical variables). Spearman’s rank correlation was analyzed by bivariate correlations. To define independent factors predicting outcome, a binary logistic regression model was used after adjusting for age, gender, WFNS score, and occurrence of DCI during hospitalization. The impact of various factors on the outcome was quantified using odds ratios (ORs) along with their corresponding 95% confidence intervals (CIs). The accuracy of GDF-15, NGAL, and ADAMTS13 in predicting unfavorable 3-month outcome and macrovascular vasospasm was evaluated by receiver operating characteristic curves, and the data are presented as the area under the curve (AUC) along with sensitivity and specificity of optimal cutoff value (calculated by Youden index). In line with current statistical consensus, an AUC of 0.8–0.9 is considered excellent, 0.7–0.8 is considered acceptable, 0.5–0.7 is considered poor in terms of accuracy of the test under consideration [48]. The cutoff of biomarkers value was also confirmed using this method. All statistical analyses were performed using statistical software (SPSS Statistics v22.0; IBM Corp., Armonk, NY, USA. The level of significance was set at 0.05 (two-sided). Graphical presentation was performed with GraphPad Prism software (GraphPad Software Version 9, San Diego, CA, USA).

## Figures and Tables

**Table 1:** Demographic profile and outcome in study population.

Variable	Patients with SAH
*N*	150
Age (years), mean ± SD	55 ± 12
Gender, female, numbers (% of total)	104 (69)
Hypertension, numbers (% of total)	71 (47)
NIDDM, numbers (% of total)	16 (11)
Smoking, numbers (% of total)	28 (19)
modified Fischer score, median, IQR	3 (2–3)
WFNS score, numbers (% of total)	
I	59 (39)
II	33 (22)
III	11 (7.3)
IV	10 (6.7)
V	37 (25)
Multiple aneurysms, numbers (% of total)	43 (29)
Aneurysm location, numbers (% of total)	
ICA	18 (12)
MCA	39 (26)
ACoA	51 (34)
PCom	12 (8)
ACA	11 (7)
VB	19 (13)
Ventricular drain, numbers (% of total)	68 (45)
Lumbar drain, numbers (% of total)	57 (38)
Decompressive craniotomy, numbers (% of total)	18 (12)
Mechanical ventilation, numbers (% of total)	66 (44)
CSF infection, numbers (% of total)	14 (9)
Systemic infection, numbers (% of total)	33 (22)
Sonologic vasospasm, numbers (% of total)	56 (37)
Onset time of sonologic vasospasm, days, median (IQR)	6.5 (4–9)
Macrovascular vasospasm, numbers (% of total)	41 (27)
Onset time of macrovascular vasospasm, day, median (IQR)	8 (6–9)
Delayed cerebral ischemia, numbers (% of total)	34 (23)
Onset time of DCI, day, median (IQR)	8 (5–11)
Favorable outcome (Day 90), numbers (% of total)	70 (47)

Values are expressed as numbers (% of total), mean ± SD or median (IQR). SAH, subarachnoid hemorrhage; N, number; NIDDM, non-insulin-dependent diabetes mellitus; WFNS score, World Federation of Neurosurgical Societies score; ICA, internal carotid artery; MCA, middle cerebral artery; ACoA, anterior communicating artery; PCom, posterior communication artery; ACA, anterior cerebral artery; VB, vertebrobasilar; CSF, cerebrospinal fluid; IQR, interquartile range; DCI, delayed cerebral ischemia.

**Table 2:** Serum level of ADAMTS13, GDF-15, and NGAL according to admission severity of SAH.

Marker/Time Points	WFNS I-III, *n* = 102			WFNS IV-V, *n* = 48			
	Median, ng/mL	Interquartile Range		Median, ng/mL	Interquartile Range		*p*-Value
ADAMTS13/D1	2589.68	1798.74	3078.70	2383.22	1918.90	2871.88	0.206
ADAMTS13/D3	2530.23	1920.05	3154.64	2190.62	1834.78	2660.88	0.184
ADAMTS13/D5	2573.34	1965.41	3026.97	2480.44	1837.69	3135.82	0.622
ADAMTS13/D7	**2632.80**	**2019.50**	**3209.94**	**2198.12**	**1598.33**	**2730.10**	**0.038**
ADAMTS13/D9	2660.20	2162.93	3319.75	2224.08	1695.33	3077.56	0.091
	P_trend_ = 0.747			P_trend_ = 0.867			
GDF15/D1	**2.99**	**1.93**	**4.85**	**4.64**	**3.45**	**7.40**	**0.001**
GDF15/D3	**2.92**	**1.62**	**4.68**	**4.35**	**3.94**	**6.29**	**<0.001**
GDF15/D5	**2.54**	**1.84**	**3.86**	**6.04**	**3.77**	**16.04**	**<0.001**
GDF15/D7	**2.41**	**1.63**	**3.92**	**7.33**	**5.10**	**14.26**	**<0.001**
GDF15/D9	**2.74**	**1.66**	**3.87**	**6.28**	**4.60**	**13.53**	**<0.001**
	P_trend_ = 0.919			P_trend_ = 0.208			
NGAL/D1	462.23	266.20	721.58	534.41	373.04	719.52	0.230
NGAL/D3	350.13	229.53	708.22	496.44	251.35	768.72	0.315
NGAL/D5	331.34	235.64	557.28	389.24	288.07	705.22	0.180
NGAL/D7	407.84	249.49	581.54	490.46	368.16	788.13	0.068
NGAL/D9	420.86	302.60	754.04	494.53	360.65	724.72	0.543
	P_trend_ = 0.284			P_trend_ = 0.799			
	mFisher score I-II, *n* = 51			mFisher score III-IV, *n* = 99			
ADAMTS13/D1	**1988.69**	**1601.32**	**2589.68**	**2823.11**	**1941.26**	**3008.73**	**0.014**
ADAMTS13/D3	**1972.90**	**1608.09**	**2565.11**	**2633.11**	**1921.11**	**3218.44**	**0.018**
ADAMTS13/D5	2310.33	1824.31	2693.09	2563.74	1911.39	3116.20	0.162
ADAMTS13/D7	2144.63	1742.80	2613.93	2576.12	1820.17	3209.94	0.252
ADAMTS13/D9	2367.95	2084.82	3204.92	2422.68	1705.39	3296.32	0.755
	P_trend_ = 0.057			P_trend_ = 0.949			
GDF15/D1	**2.43**	**1.76**	**3.90**	**3.79**	**2.28**	**6.82**	**0.004**
GDF15/D3	**3.00**	**1.39**	**3.97**	**4.09**	**2.56**	**5.50**	**0.012**
GDF15/D5	**2.13**	**1.39**	**3.18**	**3.77**	**2.65**	**10.32**	**<0.001**
GDF15/D7	**1.76**	**1.20**	**2.67**	**5.11**	**3.07**	**9.74**	**<0.001**
GDF15/D9	**2.38**	**1.61**	**3.62**	**4.37**	**2.48**	**10.78**	**<0.001**
	P_trend_ = 0.436			P_trend_ = 0.376			
NGAL/D1	**309.04**	**188.97**	**462.23**	**566.08**	**380.50**	**877.28**	**<0.001**
NGAL/D3	**252.36**	**149.78**	**342.94**	**505.36**	**280.46**	**1176.92**	**0.001**
NGAL/D5	298.72	216.70	410.36	374.22	245.35	705.22	0.072
NGAL/D7	340.73	253.22	538.52	434.01	287.65	612.29	0.097
NGAL/D9	398.80	272.03	668.35	477.61	299.67	672.98	0.738
	P_trend_ = 0.750			P_trend_ = 0.106			

SAH, subarachnoid hemorrhage; WFNS score, World Federation of Neurosurgical Societies score; mFisher score, modified Fisher score; GDF-15, growth differentiation factor-15; n, number; D, day; F-D90, favorable 3-month outcome; UF-D90, unfavorable 3-month outcome; significant *p*-values are highlighted in bold. P_trend_ = indicates whether groups diverged differently over time.

**Table 3:** Binary logistic regression analysis of associations of GDF-15 with unfavorable outcome at 3 months.

Parameter	*p* Value	Unadjusted OR (95% CI)	*p* Value	Adjusted OR (95% CI) ^†^
GDF-15/D1	0.001	1.456 (1.159–1.830)	0.015	1.895 (1.132–3.172)
GDF-15/D3	<0.001	1.848 (1.339–2.549)	0.008	1.599 (1.129–2.267)
GDF-15/D5	<0.001	1.802 (1.326–2.449)	0.005	1.778 (1.195–2.645)
GDF-15/D7	<0.001	1.806 (1.362–2.394)	0.007	1.587 (1.137–2.216)
GDF-15/D9	0.001	1.681 (1.237–2.283)	0.042	1.512 (1.016–2.251)

OR, odd ratio; CI, confidential interval; GDF-15, growth differentiation factor-15; D, days; ^†^ adjusted for: age, gender, WFNS, occurrence of DCI.

**Table 4:** ROC analysis for GDF-15 levels associated with unfavorable outcome (mRS ≥ 3) at 3 months.

	Cut-Off Value *	AUC	*p*-Value	95% CI		Sensitivity (%)	Specificity (%)	Power
				Lower Limit Upper Limit				
Total cohort								
GDF-15 D1	3.16	0.728	<0.001	0.627	0.830	76.1	61.7	acceptable
GDF-15 D3	3.93	0.844	<0.001	0.765	0.924	78.6	81.2	excellent
GDF-15 D5	2.77	0.865	<0.001	0.790	0.940	89.1	70.8	excellent
GDF-15 D7	3.59	0.847	<0.001	0.764	0.930	82.2	82.6	excellent
GDF-15 D9	3.06	0.809	<0.001	0.711	0.907	78.4	66.7	excellent
WFNS I-II-III								
GDF-15 D1	3.31	0.669	0.037	0.519	0.819	66.7	64.4	poor
GDF-15 D3	3.97	0.833	<0.001	0.728	0.938	73.7	82.6	excellent
GDF-15 D5	3.06	0.783	<0.001	0.657	0.909	75	78.7	acceptable
GDF-15 D7	2.41	0.699	0.015	0.538	0.859	66.7	56.8	poor
GDF-15 D9	3.06	0.696	0.017	0.542	0.851	66.7	65.9	poor

AUC, area under the curve; CI, confidential interval; WFNS, World Federation of Neurosurgical Societies score; GDF-15, growth differentiation factor-15; D, days; mRS, modified Rankin score; * ng/mL; power, ability to diagnose patients with and without the disease or condition based on the test, AUC of 0.8–0.9 is considered excellent, 0.7–0.8 is considered acceptable, 0.5–0.7 is considered poor in terms of accuracy of the test under consideration.

**Table 5:** ROC analysis for serum NGAL and ADAMTS13 values associated with macrovascular vasospasm (*n* = 41) during hospitalization in patients with aneurysmal subarachnoid hemorrhage.

	Cut-Off Value *	AUC	*p*-Value	95% CI		Sensitivity (%)	Specificity (%)	Power
				Lower Limit Upper Limit				
NGAL/D1	453	0.709	0.001	0.590	0.829	82.1	56.9	acceptable
NGAL/D3	518	0.776	<0.001	0.665	0.887	78.3	73.1	acceptable
NGAL/D5	368	0.719	0.001	0.601	0.838	74.1	61.2	acceptable
ADAMTS13/D7	2236	0.701	0.003	0.574	0.827	68.2	64	acceptable
ADAMTS13/D9	2276	0.676	0.024	0.525	0.827	68.9	72.2	poor

AUC, area under the curve; CI, confidential interval; NGAL, neutrophil gelatinase-associated lipocalin; D, days; * ng/mL; ROC analysis for D7 and D9 of NGAL were not displayed because of its non-significance value. power, ability to diagnose patients with and without the disease or condition based on the test, AUC of 0.8–0.9 is considered excellent, 0.7–0.8 is considered acceptable, 0.5–0.7 is considered poor in terms of accuracy of the test under consideration.

**Table 6:** Association between level of NGAL and ADAMTS13 and macrovascular vasospasm, binary logistic regression analysis.

Parameter	*p* Value	Unadjusted OR (95% CI)	*p* Value	Adjusted OR (95% CI) ^†^
NGAL/D1	0.002	1.002 (1.001–1.003)	0.088	1.002 (1.000–1.003)
NGAL/D3	0.006	1.001 (1.000–1.002)	0.056	1.002 (1.000–1.004)
NGAL/D5	0.007	1.002 (1.000–1.003)	0.808	1.000 (0.997–1.004)
ADAMTS13/D7	0.004	0.999 (0.998–1.000)	0.036	0.999 (0.997–1.000)
ADAMTS13/D9	0.032	0.999 (0.999–1.000)	0.059	0.999 (0.998–1.000)

OR, odd ratio; CI, confidential interval; NGAL, neutrophil gelatinase-associated lipocalin; ADAMTS13, a disintegrin and metalloproteinase with a thrombospondin type 1 motif member 13; D, days; ^†^ adjusted for: age, gender, modified Fisher score at admission CT and occurrence of systemic infection during hospitalization.

## Data Availability

The datasets used and or analyzed in the current study are available from the corresponding author upon reasonable request.

## Supplementary Materials

The following supporting information can be downloaded at: https://www.mdpi.com/article/10.3390/ijms241311005/s1.
