# Supplementary material for: Different Kinetics of Serum ADAMTS13, GDF-15, and Neutrophil Gelatinase-Associated Lipocalin in the Early Phase of Aneurysmal Subarachnoid Hemorrhage
Source: Int J Mol Sci. 2023 Jul 2;24(13):11005. doi: 10.3390/ijms241311005 (PMC10341640; doi:10.3390/ijms241311005)
Supplement: Supplementary file 1 [file ijms-24-11005-s001.zip › ijms-2473073-supplementary.pdf]

Table S1. Serum levels of ADAMTS13, GDF-15 and NGAL according to 3-month functional outcome.

	Favorable at D90 (n=70)			Unfavorable at D90 (n=80)			
	Median	Interquartile range		Median	Interquartile range		p-value
ADAMTS13 /D1	2747,4	1826,4	3078,7	2352,9	1798,7	2897,0	0.213
ADAMTS13 /D3	2247,2	1829,5	2954,1	2431,7	1869,9	2959,0	0.525
ADAMTS13 /D5	2558,3	2052,1	2878,9	2617,0	1927,2	3153,1	0.356
ADAMTS13 /D7	2522,4	2002,9	2931,5	2574,5	1820,2	3121,6	0.742
ADAMTS13 /D9	2663,9	2130,3	3616,8	2341,8	2041,9	3044,2	0.188
	P_{trend}=0.254			P_{trend}=0.268			
GDF-15 /D1	2,6	1,9	4,7	4,3	3,2	6,9	<0.001
GDF-15 /D3	2,4	1,4	3,8	4,8	4,0	6,3	<0.001
GDF-15 /D5	2,3	1,5	3,0	5,0	3,5	10,0	<0.001
GDF-15 /D7	2,2	1,6	3,2	6,2	3,9	10,5	<0.001
GDF-15 /D9	2,4	1,6	3,5	5,3	3,1	11,1	<0.001
	P_{trend}=0.950			P_{trend}=0.265			
NGAL /D1	430,3	250,4	696,4	513,0	360,9	721,6	0.144
NGAL /D3	306,3	207,1	686,0	516,9	264,3	881,4	0.086
NGAL /D5	304,6	222,7	537,6	380,4	315,7	705,2	0.049
NGAL /D7	382,2	253,2	538,5	513,2	312,4	697,9	0.030
NGAL /D9	398,5	299,0	581,6	500,3	370,5	818,7	0.101
	P_{trend}=0.431			P_{trend}=0.771			

ADAMTS13 a disintegrin and metalloproteinase with a thrombospondin type 1 motif, member 13, *NGAL* neutrophil gelatinase-associated lipocalin, *GDF-15* Growth differentiation factor-15, *Favorable D90* modified Rankin score at 3-month follow-up: 0-2, *Unfavorable at D90* modified Rankin score at 3-month follow-up: 3-6

Table S2. Serum levels of GDF-15, NGAL and ADAMTS13 in different groups of macrovascular vasospasm and delayed cerebral ischemia.

Marker /Time points	Macrovascular vasospasm negative group (n=109)						
	DCI negative, n=98			DCI positive, n=11			
	Median	Percentile 25	Percentile 75	Median	Percentile 25	Percentile 75	p-value
GDF-15 /D1	3,758	2,4035	5,174	3,6545	2,872	5,435	0.794
GDF-15 /D3	3,423	1,771	4,592	5,5085	4,68	14,422	**0.02**
GDF-15 /D5	2,708	1,952	4,98	4,7125	3,855	8,011	**0.049**
GDF-15 /D7	3,228	1,731	6,0105	5,1625	3,24	14,276	0.153
GDF-15 /D9	3,0825	1,8035	5,1965	6,241	3,079	8,064	0.186
NGAL /D1	366,9885	251,038	541,368	436,533	276,732	514,948	0.592
NGAL /D3	287,417	181,186	453,079	404,106	229,53	726,464	0.391
NGAL /D5	303,352	216,698	389,238	335,732	245,348	366,782	0.878
NGAL /D7	406,927	270,518	552,6265	430,499	234,816	558,879	0.880
NGAL /D9	398,5	299,328	618,3055	614,81	500,096	835,285	0.177

ADAMTS13 /D1	2577,727	1923,7205	3108,325	2914,528	2398,486	3416,574	0.222
ADAMTS13 /D3	2199,731	1834,775	2959,015	2807,846	2418,299	3383,25	0.087
ADAMTS13 /D5	2656,68	2044,503	3429,4215	2925,866	2198,116	3148,139	0.139
ADAMTS13 /D7	2656,68	2044,503	3429,4215	2925,866	2198,116	3148,139	0.917
ADAMTS13 /D9	2663,941	2178,969	3308,0335	3044,188	2942,367	3190,878	0.239

	Macrovascular vasospasm positive group (n=41)						
	DCI negative (n=12)			DCI positive (n=29)			
	Median	Percentile 25	Percentile 75	Median	Percentile 25	Percentile 75	p-value
GDF-15 /D1	4,1485	2,867	4,639	3,452	2,252	4,483	0.661
GDF-15 /D3	1,699	0,89	6,086	4,0815	2,794	4,863	0.469
GDF-15 /D5	2,239	1,537	5,3995	4,108	3,087	6,037	0.142
GDF-15 /D7	2,32	1,792	5,804	3,916	3,266	6,333	0.280
GDF-15 /D9	2,357	1,726	4,526	4,541	2,797	5,237	0.260
NGAL /D1	335,705	171,895	1365,2135	719,517	491,892	999,331	0.226
NGAL /D3	281,272	219,221	1196,846	649,1015	292,437	881,411	0.692
NGAL /D5	350,544	120,8295	1202,483	375,7145	331,341	691,083	0.733
NGAL /D7	237,75	168,964	664,818	648,411	513,248	988,408	0.104
NGAL /D9	275,983	176,605	879,4105	488,748	441,01	739,118	0.260
ADAMTS13 /D1	2215,062	1366,4655	3795,6405	2398,486	1941,256	2897,041	0.851
ADAMTS13 /D3	1747,751	1007,848	2758,342	2142,521	1869,904	2445,025	0.573
ADAMTS13 /D5	2159,995	1308,1355	3594,823	2357,344	1699,706	3023,792	0.988
ADAMTS13 /D7	2028,011	1843,994	3038,9195	2235,72	1598,328	2714,918	0.753
ADAMTS13 /D9	2074,699	1545,238	2502,9625	2041,863	1698,682	3252,335	0.903
